# WSMD: weakly-supervised motif discovery in transcription factor ChIP-seq data

**DOI:** 10.1038/s41598-017-03554-7

**Published:** 2017-06-12

**Authors:** Hongbo Zhang, Lin Zhu, De-Shuang Huang

**Affiliations:** Institute of Machine Learning and Systems Biology, College of Electronics and Information Engineering, Tongji University, Shanghai, 201804 P.R. China

## Abstract

Although discriminative motif discovery (DMD) methods are promising for eliciting motifs from high-throughput experimental data, due to consideration of computational expense, most of existing DMD methods have to choose approximate schemes that greatly restrict the search space, leading to significant loss of predictive accuracy. In this paper, we propose Weakly-Supervised Motif Discovery (WSMD) to discover motifs from ChIP-seq datasets. In contrast to the learning strategies adopted by previous DMD methods, WSMD allows a “global” optimization scheme of the motif parameters in continuous space, thereby reducing the information loss of model representation and improving the quality of resultant motifs. Meanwhile, by exploiting the connection between DMD framework and existing weakly supervised learning (WSL) technologies, we also present highly scalable learning strategies for the proposed method. The experimental results on both real ChIP-seq datasets and synthetic datasets show that WSMD substantially outperforms former DMD methods (including DREME, HOMER, XXmotif, motifRG and DECOD) in terms of predictive accuracy, while also achieving a competitive computational speed.

## Introduction

As the main regulators of transcription process, transcription factors (TFs) can modulate gene expression by binding to special DNA regions, which are known as TF binding sites (TFBS). Previous researches have concluded that TFs are relatively conserved in the long-term evolution, and are inclined to bind to DNA sequences that follow specific patterns, which are commonly called TFBS motifs^1–3^. Recognition of these motifs is fundamental for further understanding of the regulatory mechanisms, and is still a challenging task in computational biology^4, 5^.

In the past few decades, due to the rapid development of high-throughput sequencing technology, a variety of experimental methods have been developed to extract TF-DNA binding regions. In particular, ChIP-Seq, which combines chromatin immunoprecipitation with high-throughput sequencing, greatly improves the amount and spatial resolution of generated data, which are conducive to the studies of modeling TF binding *in vivo* ^6, 7^. However, ChIP-Seq also brings two challenges for motif discovery methods: (i) The enormous amount of potential TF binding regions yielded from a single experiment requires highly scalable motif discovery (MD) tools^8, 9^; (ii) The large quantities of datasets also increase the possibility of finding multiple enriched sequence features, and most of them may either be false positives or not directly related to the problem of interest, which make it necessary for MD tools to be capable of understanding the nature of motif signals and determining the relevant ones^10–12^.

Currently, many algorithms tailored for high-throughput datasets have been proposed^13–15^. Among existing approaches, the discriminative motif discovery (DMD) methods offer a promising strategy for simultaneously addressing the aforementioned two challenges^16–18^. Similar to traditional MD methods, DMDs treat the peak regions of ChIP-seq dataset as foreground sequences, where the motif instances are assumed to be statistically enriched; unlike traditional methods, however, they represent non-binding regions in the foreground using some carefully selected background sequences, then reformulate MD as the extraction of sequence features which could discriminate the foreground sequences from background sequences.

Computationally, the learning of DMDs is more difficult than general discriminant tasks encountered in machine learning: on one hand, the learner not only needs to correctly classify the sequences as foreground or background, but also has to locate the binding sites in foreground examples; on the other hand, the learning objectives of DMDs are generally nonconvex, nondifferentiable, and even discontinuous, and are thus difficult to optimize. To circumvent such difficulties and improve scalability, current DMD methods typically do not search for motif directly over the complete parameter space, but instead adopt approximate schemes that could sacrifice both accuracy and expressive power. For example, the motifs learned by DREME^19^ and motifRG^18^ are limited to the discrete IUPAC space, while HOMER^20^ and XXmotif^21^ choose to refine motifs by only tuning some external parameters.

Meanwhile, in the computer vision community, object detection (OD) is an important and quite challenging application: Given a set of positive images that contain the object of interest, and another set of negative images that don’t contain the object, OD aims to classify the test images accurately as positive or negative, and locate the objects of interest in positive images simultaneously. Such kind of problems is also called weakly supervised learning (WSL) in the machine learning community, and various successful techniques have been proposed therein^22–26^.

**Figure 1:**
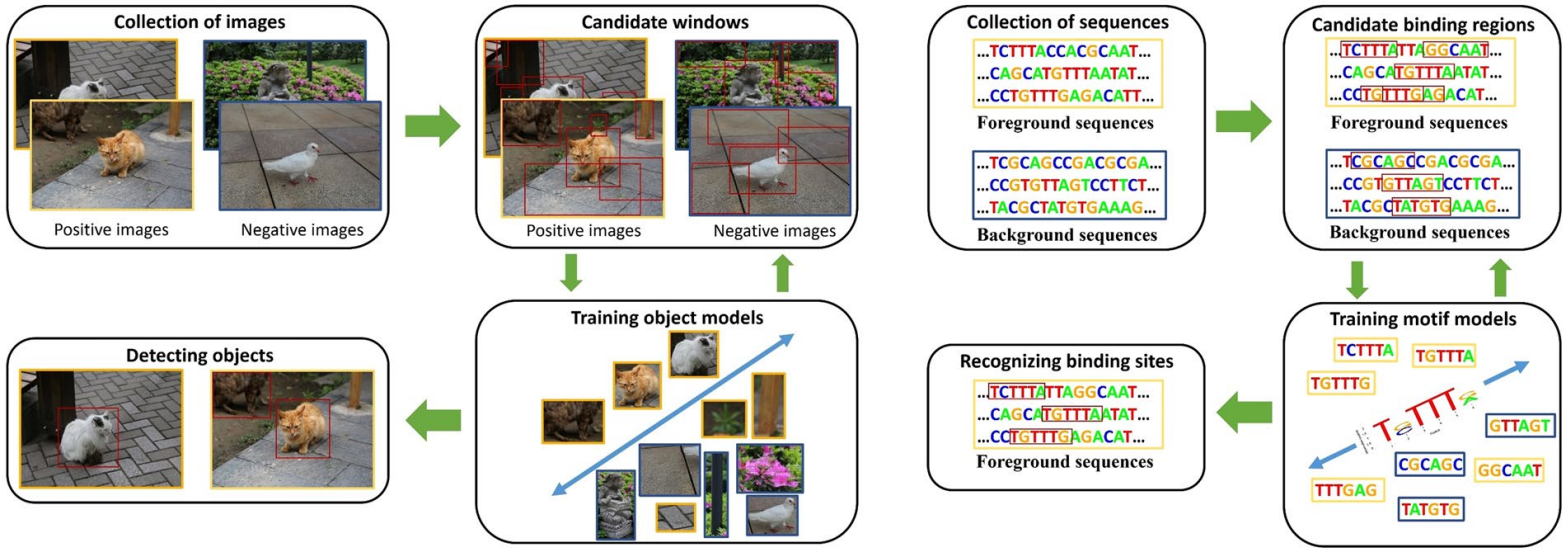
An overview of object detection and discriminative motif discovery. (**a**) Object detection. (**b**) Discriminative motif discovery.

The DMD task shares many features with OD: we know that TF binds to specific sites in ChIP-seq enriched regions, but we don’t know exactly where, just as we don’t know the exact location of the object of interest in a positive image. In addition, the framework of OD generally consists of four steps (Fig. 1(a)): (1) Collection of training images; (2) Generation of candidate windows that are likely to include the considered object; (3) Learning the object models and refining the candidate windows iteratively with some WSL technologies; (4) Detecting the windows which contain the object with the optimal object models. Similar to OD, DMD also generally consists of four corresponding steps (Fig. 1(b)) which take collection of foreground and background DNA regions as input sequences, then identify the relevant motif by alternating between extracting candidate binding regions and training motif model, finally recognize the real binding sites with the reported motif. Due to these apparent similarities between DMD and OD, and the excellent performance of WSL in OD, it seems natural to integrate the WSL technologies into DMD framework to address the challenges brought by high-throughput ChIP-seq datasets.

As a first attempt to combine the DMD framework with the WSL technologies, in this paper we propose a novel MD approach named WSMD (Weakly-Supervised Motif Discovery) to identify motifs from ChIP-seq datasets. Firstly, we propose to learn the optimal motifs by maximizing classification accuracy (CA), which is one of the most popular metrics in pattern recognition. Similar to other widely used metrics in DMDs, CA is also based on the contingency table; meanwhile, CA has the additional advantage that it can be easily reformulated as a continuous function using convex surrogates. Then, we show that the resulting optimization problem is essentially equivalent to latent support vector machine (LSVM), a widely studied formulation in OD. With this inherent similarity, many WSL learning strategies, which have excellent performances in OD, can be utilized to solve this optimization task. In contrast to the learning strategies adopted by previous DMD methods, WSMD allows “global” optimization of PWMs in continuous space, thereby reducing the information loss of model representation and improving the quality of resultant motifs. Finally, we compare the performance of WSMD with five well-known DMD methods (DREME, XXmotif, HOMER, motifRG and DECOD). The experimental results on 134 real ChIP-seq datasets show that the motifs found by WSMD have better statistical significance, as measured by three commonly used evaluation criteria (AUC under ROC, Fisher’s exact test score and the Minimal Hyper-Geometric score). Further in-depth experiments on two groups of synthetic datasets also show that the individual steps of our method have advantages over the five benchmarked methods. The R package of our algorithm is available at https://github.com/hongbozhang0808/WSMD.

## Methods

### Overview of motif discovery

Motif discovery is one of the most studied branches in bioinformatics, and the existing literature is vast. Here we give a brief overview of the related works and recommend the interested authors to^13, 17, 27^ for detailed reviews. Based on the model representation, motif discovery algorithms can be generally classified as ‘word-based’ or ‘probabilistic-based’^28^. Word-based algorithms model TF binding affinities with consensus sequence, which represents the predominant nucleotide at each site with IUPAC ambiguity codes^19, 29, 30^. On the other hand, probabilistic-based algorithms generally perform local searches for the most represented segments in input sequences and represent them as probabilistic models. One of the most commonly used probabilistic models is Position Weight Matrix (PWM)^8, 31, 32^. Compared with consensus sequence, PWM is a more nuanced representation of motif. It models TF binding affinities with a 4 × *l* matrix which describes binding affinities as probability distributions over DNA alphabet.

Computationally, DMDs typically search an extremely large space of candidate motifs to look for the motif that maximizes an “objective function” which quantifies the degree of discriminability. Naturally, the choice of objective functions would significantly affect the quality of elicited motifs. In practice, the objective functions of DMDs are generally built upon the statistical features of input sequences, and one of frequently considered features is the statistic that describes whether a sequence contains a target motif. Based on this statistic, we can construct a contingency table (Table 1) which tabulates the number of foreground/background sequences that contain or don’t contain a motif instance. Many objective functions of DMDs are defined using the contingency table^33^, such as the Fisher’s exact test score adopted by DREME and SeAMotE^16^ and the relative frequency difference used by DECOD^8^ and DIPS^34^.

**Table 1:** Contingency table.

	Motif present	Motif absent
Foreground	*TP*	*FN*
Background	*FP*	*TN*

Here *TP* and *FP* stand for true and false positives, *TN* and *FN* for true and false negatives, respectively.

Aside from being able to accurately measure the motif discriminability, the learning objectives of DMDs should also allow for efficient and effective optimization, which are especially important when dealing with high-throughput datasets. While the aforementioned contingency-table-based metrics all could reasonably quantify the “discrimination score” of motifs, they are generally nonconvex, nondifferentiable, and even discontinuous, and are thus difficult to optimize numerically. To alleviate such difficulties, a variety of heuristic searching procedures were applied in the previously mentioned DMD methods. For example, DREME and motifRG greedily refine the initial motifs in site-by-site manner, and restrict the search for motifs to the discrete IUPAC space. On the other hand, despite differences in implementation details, HOMER and XXmotif essentially adopt the same strategy: they iteratively tweak the site score threshold so as to maximize the motif enrichment in foreground versus background sequences, then update the PWMs using *k*-mers scored above the selected detection threshold. Although such strategies could provide PMW-based motif representations to avoid the drawbacks of DREME and motifRG, they are still limited since only one indirect motif parameter (i.e., the detection threshold) is optimized.

Here, we model the transcription factor binding specificities with PWMs and adopt the classification accuracy (CA) as the learning objective, which measures the proportion of true predicted results (both *TP* and *TN*) among the total number of sequences. Similar to the aforementioned metrics such as Fisher exact test score, CA is also based on contingency table. Besides, as a widely used statistical measure in pattern classification, CA has the additional advantage that it can be easily reformulated as a continuous function using convex surrogates. Meanwhile, as will be detailed later, the resulting optimization problem is essentially equivalent to latent support vector machine (LSVM), which is widely studied in the OD literatures. Furthermore, by exploiting this connection, efficient learning scheme can be designed to solve the proposed optimization task.

### CA-based discriminative object function

We used unified notations in this paper. Lower case italic letters such as *x* denote scalars, bold lower case letters represent vectors, such as **x** = (*x*_*i*_) ∈ R_*m*_. Bold upper case letters denote matrices, such as **X** = (*x*_*ij*_) ∈ R_*m* × *n*_, and bold upper case italic letters represent vector sets, such as ***X*** = (**x**_*i*_), **x**_*i*_ ∈ R_*m*_. |**x**| and |***X***| return the size of vector **x** and ***X*** respectively.

We would like to solve the following problem: Given ***F*** and ***B*** as foreground and background sequence set, respectively, our task is to learn a motif, represented by PWM **P** ∈ R_4__×__*l*_, which can distinguish ***F*** from ***B*** with the maximal CA. Formally, the object function can be written as follows

1 $$\mathop{\max }\limits_{{\bf{P}}}(\frac{TP\,+\,TN}{TP\,+\,FN\,+\,TN\,+\,FP}),$$

which is equivalent to the minimization of classification error:

2 $$\mathop{\max }\limits_{{\bf{P}}}(\frac{TP\,+\,TN}{TP\,+\,FN\,+\,TN\,+\,FP})\,\iff \,\mathop{\min }\limits_{{\bf{P}}}\frac{1}{|{\boldsymbol{F}}|\,+\,|{\boldsymbol{B}}|}(FN\,+\,FP).$$

For word-based MD methods, the calculation of (2) is easy since the occurrence of the motif is well defined. For probabilistic-based methods such as ours, an additional site score threshold *b* is required, and whether a sequence contains a motif is defined based on whether it contains a site scored above the threshold^35^. Formally, *FN* and *FP* in (2) can be defined as follows

3 $$\{\begin{array}{l}FN\,=\,\sum \mathrm{sgn}\,({\rm{E}}\,({\bf{P}},{{\bf{s}}}_{i})\,-\,b\, < \,0)\,,\,{{\bf{s}}}_{i}\,\in \,{\boldsymbol{F}}\\ FP\,=\,\sum \mathrm{sgn}\,({\rm{E}}\,({\bf{P}},{{\bf{s}}}_{i})-b > 0)\,,\,{{\bf{s}}}_{i}\,\in \,{\boldsymbol{B}}\end{array},$$

where sgn (·) is an indicator function which returns 1 if the argument is true and 0 otherwise. Let ***S*** be the set of all possible *l*-mers in sequences **s**, E (**P**,**s**) returns the maximal binding energy of all elements in ***S*** with motif **P**:

4 $${\rm{E}}({\bf{P}},\,{\bf{s}})\,=\,\max ({\rm{E}}({\bf{P}},\,{{\bf{s}}}^{{\rm{sub}}}),{{\bf{s}}}^{{\rm{sub}}}\,\in \,{\boldsymbol{S}}),$$

where the site-level binding energy is defined as

5 $${\rm{E}}({\bf{P}},\,{{\bf{s}}}^{{\rm{sub}}})\,=\,\sum _{i=1}^{l}\mathrm{log}({{\bf{P}}}_{(i,{({{\bf{s}}}^{{\rm{sub}}})}_{i})}).$$

The formulation in (5) can be further simplified: for a given *l*-length DNA sequence **s** ^sub^, we can encode it as a *4l*-length binary feature vector **x** ^sub^ by transforming each nucleotide into a 4-dimensional vector using “one-hot encoding”:

6 $$\{\begin{array}{c} \textquotedbl {\rm{A}}\, \textquotedbl \,=\,\{1,0,0,0\}, \textquotedbl {\rm{C}} \textquotedbl \,=\,\{0,1,0,0\},\\  \textquotedbl {\rm{G}} \textquotedbl \,=\,\{0,0,1,0\}, \textquotedbl {\rm{T}} \textquotedbl \,=\,\{0,0,0,1\}.\end{array}$$

We can also convert the logarithm of **P** to a *4l*-length vector **w** by concatenating its column vectors as a single vector. Then the binding energy (5) can be evaluated as the inner production of **w** and **x** ^sub^:

7 $$E\,({\bf{P}},{{\bf{s}}}^{{\rm{sub}}})\,=\,{{\bf{w}}}^{T}{{\bf{x}}}^{{\rm{sub}}}.$$

By applying Equations (3), (4) and (7) to(2), the objective function is rewritten as

8 $$\mathop{\min }\limits_{{\bf{w}},b}\,\frac{1}{|{\boldsymbol{F}}|+|{\boldsymbol{B}}|}\sum _{{\bf{s}}\,\in \,{\boldsymbol{F}}\cup {\boldsymbol{B}}}\mathrm{sgn}\,({y}_{{\bf{s}}}\,(\mathop{\max }\limits_{{{\bf{s}}}^{{\rm{sub}}}\,\in \,{\boldsymbol{S}}}({{\bf{w}}}^{T}{{\bf{x}}}^{{\rm{sub}}})-b) < 0),$$

where y_s_ = 1 if **s** **∈** ***F*** and −1 otherwise.

Numerically, the indicator function sgn(*x*) is still non-convex and its optimization is NP-hard in general, thus most works in the machine learning literatures replace the indicator function with a convex upper bound that has better computational guarantees^36^. Here we specifically choose the hinge function^37^, which can be defined as follows

9 $$\text{hinge}\,(x)\,=\,\max \,(0,\,1-x).$$

Additionally, we add an L2 norm penalty term ${\Vert {\bf{w}}\Vert }_{2}^{2}$ to the objective function to avoid overfitting, and rewrite Equation (8) as

10 $$\mathop{\min }\limits_{{\bf{w}},b}{\Vert {\bf{w}}\Vert }_{2}^{2}+\frac{c}{|{\boldsymbol{F}}|+|{\boldsymbol{B}}|}\sum _{{\bf{s}}\,\in \,{\boldsymbol{F}}\,\cup \,{\boldsymbol{B}}}\max (1-{y}_{{\bf{s}}}\,(\mathop{\max }\limits_{{{\bf{s}}}^{{\rm{sub}}}\,\in \,{\boldsymbol{S}}}({{\bf{w}}}^{T}{{\bf{x}}}^{{\rm{sub}}})-b),0),$$

where *c* controls the tradeoff between the classification error rate and the complexity of training model. By introducing a slack variable *ξ*_*i*_ for each sequence *s*_*i*_ ∈ ***F*** **∪** ***B***, we can transform (10) into a less convoluted form as

11 $$\begin{array}{c}\mathop{\min }\limits_{{\bf{w}},{\boldsymbol{\xi }},b}{\Vert {\bf{w}}\Vert }_{2}^{2}+\frac{c}{|{\boldsymbol{F}}|+|{\boldsymbol{B}}|}\sum {\xi }_{i},\\ {\rm{s}}{\rm{.t}}{\rm{.}}\,{y}_{{\bf{s}}}(\mathop{\max }\limits_{{{\bf{s}}}^{{\rm{sub}}}\in {\boldsymbol{S}}}({{\bf{w}}}^{T}{{\bf{x}}}^{{\rm{sub}}})-b)+{\xi }_{i}\ge 1,\,{\xi }_{i}\ge 0,\,\forall \,{\bf{s}}\in {\boldsymbol{F}}\cup {\boldsymbol{B}}.\end{array}$$

The above-mentioned objective function is analogous to the classical latent SVM (LSVM), which is a widely used formulation of WSL in OD^22^. There, instead of DNA sequences, the input foreground and background datasets are labeled images, and **w** could be regarded as a vectorized “template” which describes the object of interest; on the other hand, while in motif learning, the latent variable **s** ^sub^ represents the most potential binding site in sequence, in OD it instead represents the sub-region in a picture that most resembles the object to be detected.

As will be detailed later, this LSVM-based formulation of DMD simultaneously provides three advantages over the existing DMD methods reviewed in the previous sections:

- Unlike the discrete searching space adopted in DREME and motifRG, formulation (11) directly learns PWMs in a continuous space, thus it is anticipated that the information loss of model representation is reduced and the quality of resultant motifs is improved.
- Compared with the greedy approach used by DREME and motifRG, and the indirect refinement strategy applied by HOMER and XXmotif, this formulation allows “global” optimization of PWM by taking all the positions of PWM into account at the same time.
- Additionally, there are a wide spectrum of existing literatures in the WSL domain that focus on developing efficient solver for LSVM, which can be adapted to obtain high-quality solutions efficiently for (11).

### Weakly-supervised motif discovery (WSMD)

Based on the formulation (11), in this section we outline the basic framework of WSMD, which can be divided into 5 stages:

#### Preprocessing

We split each input sequence and its reverse-complement with an *l*-length sliding window to obtain a bag of *l*-mers, where *l* is the desired motif length. Then we encode each *l*-mer as a feature vector with (6) to formulate each foreground/background sequence as a positive/negative set of feature vectors.

#### Seeding

We start by enumerating all exact words (without wildcards) of a given length *k* (*k* = 6 by default), then calculate the substring minimal distance (SMD)^38^ between every pair of word and input sequences. Here, SMD is defined as the minimal Hamming Distance (HD) between a word and the subsequences of an input sequence. The “discrimination score” of each word is then calculated as the probability that it has a smaller SMD in a foreground sequence than in a background. Then, the *k*-mers with the highest discrimination scores are retained for further optimization.

#### Refinement

In the refinement step, WSMD takes the top-scored *k*-mers from the Seeding stage as input, and optimizes them using(11). Although at first appearance, Equation (11) is a complex-formed nonconvex optimization problem under large number of constraints, we can still solve it efficiently by using a simple coordinate-descent-style LSVM optimization strategy^22^. The core idea is to exploit the fact that if the latent variables that mark the bound regions of each input sequences are given, the problem (11) reduces to a convex quadratic programming (QP) which can be solved efficiently using off-the-shelf software such as Mosek and CPLEX. In summary, the PWM is optimized iteratively with two alternating steps: **Update-step:** Update the TF binding regions for both foreground and background sequences using the current PWM; **QP-step:** Solve the resultant QP problem to update PWM. This procedure is repeated until the objective function value converges.

#### Extension

Generally, the seed length *k* is smaller than the desired motif length *l*. In order to extend the refined motif to length *l*, we firstly add uniform weights at *x* positions upstream and *l*−*k*−*x* positions downstream of the motif respectively, where *x* varies between 0 and *l*−*k*. Such a protocol yields *l* − *k* + 1 initial PWMs of length *l*, which are then again optimized using Equation (11), and the one that achieves the minimal objective function value is reported as the final motif.

#### Masking

In practice, the input dataset often contains multiple motifs, and often each motif explains a subset of the data^39^, which requires motif finders to be capable of extracting multiple non-redundant motifs from one dataset. To fulfill this requirement, a commonly adopted strategy in existing DMD methods is to mask the “most potential” binding regions in foreground sequences for the reported motifs, and then repeat search procedure to find other motifs. In WSMD, this can be done by simply removing corresponding feature vectors from the positive set.

Additional details about WSMD are presented in Supplementary Section 1.

## Results

In this section, the performance of WSMD is systematically evaluated by comparing it with five widely used DMD algorithms, including DREME, HOMER, XXmotif, motifRG and DECOD. We first conducted an experiment on a comprehensive collection of real ChIP-Seq datasets to show that the performance of WSMD is superior or competitive w.r.t. the other methods. Then, with further experiments on synthetic datasets, we performed in-depth analysis of the refinement and extension strategies of WSMD respectively. At last, we compared the running time of four DMD methods.

### Performance comparison on real data

To assess WSMD on real data under different conditions, we collected 134 ChIP-seq datasets from ENCODE (see Supplementary Section 2 for the complete list). For each ChIP-seq dataset, 2000 top ranking peaks were chosen as foreground sequences. On the other hand, the choice of background sequences can significantly affect the results of DMDs. It is widely recognized that the background sequences have to be selected to match the statistical properties of the foreground set^29, 40–42^, otherwise the elicited motifs could be biased. To achieve this, a commonly adopted strategy in the literature is to generate artificial background sequences based on the statistical features of foreground sequences, however, previous studies have demonstrated that such sequences are relatively “easy” negatives and could result in underestimation of false-positive rates^43^. Following^18, 43^, we instead obtained a background sequence for each peak by randomly choosing a sequence of the same length and lies 0–200 nt from the edge on either up or down strand.

**Figure 2:**
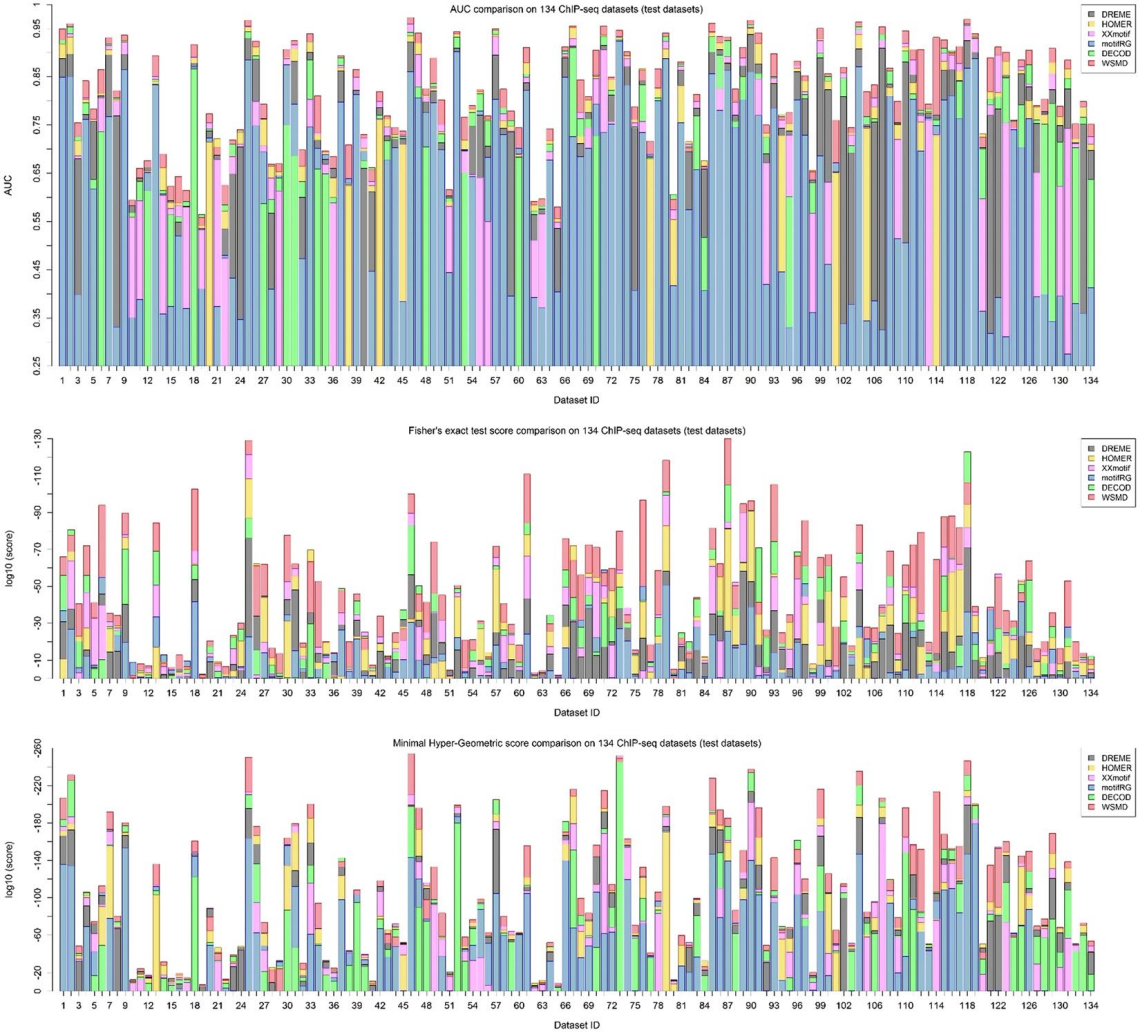
3-fold cross-validation test performance on three reference-free evaluation criteria over 134 datasets. The performances of six methods on same dataset were plotted on one horizontal bar while differing in colors. In this way, the lines with different colors in one horizontal bar present the performance archived by corresponding tools, and the height of box with different colors can show the performance improvement of corresponding tools compared with the one performing more poorly. (**a**) 3-fold cross-validation test performance on AUC over 134 datasets. (**b**) 3-fold cross-validation test performance on Fisher’s Exact Test score over 134 datasets. (**c**) 3-fold cross-validation test performance on Minimal Hyper-Geometric score over 134 datasets.

Here, 3-fold cross-validation was used as the performance evaluation scheme. In other words, for each ChIP-seq dataset we took the corresponding set of positive/negative sequences and partitioned them into 3 sets (“folds”) of roughly equal size, a PWM was trained on two folds and then evaluated on the other fold. One of the most intuitive approach for assessing DMD methods is to evaluate the similarity between predicted motifs and the reference motifs retrieved from dedicated databases^44–46^. However, this evaluation protocol is problematic since existing motif catalogs are still incomplete and may contain errors. In addition, many TFs could bind DNA cooperatively as heterodimers that alter their respective binding specificity^47^, yet motifs of these heterodimers still remain underrepresented in current motif repositories^48^. Similar to^9, 21^, we instead adopt the following reference-free metrics:

Firstly, the AUC (the area under the receiver operating characteristic curve), a widely used evaluation criterion in both machine learning and motif discovery^18, 27, 49, 50^, was evaluated to gauge and compare discriminating power of different motifs reported by four DMD methods. Figure 2(a) summarizes the average test AUC performance of four tools on 134 datasets. It is evident that WSMD almost always achieves the best discriminability on test datasets in comparison with other methods. Additionally, the paired t-test and Wilcoxon signed-rank test between WSMD and the other methods were conducted to quantify the advantages of WSMD in test AUC (Table 2 rows 1–3), and the average training and test AUC on all the 134 datasets for all the algorithms were also reported (Table 3 rows 1–3). As them shows, WSMD have a considerable advantage over other five methods.

**Table 2:** The paired t-test and Wilcoxon signed-rank test P-value between WSMD and the other methods on different evaluation criteria.

On AUC WSMD w.r.t.	DREME	HOMER	XXmotif	motifRG	DECOD
Paired t-test P-value	1.85e-42	3.19e-23	2.61e-24	9.19e-30	1.61e-23
Wilcoxon signed-rank test P-value	5.30e-24	9.08e-23	5.80e-24	4.96e-24	2.07e-23
On Fisher’s score WSMD w.r.t.	DREME	HOMER	XXmotif	motifRG	DECOD
Paired t-test P-value	3.79e-34	5.25e-21	7.31e-23	2.26e-29	5.78e-18
Wilcoxon signed-rank test P-value	7.77e-24	9.18e-22	7.14e-23	1.74e-23	3.12e-19
On MHG score WSMD w.r.t.	DREME	HOMER	XXmotif	motifRG	DECOD
Paired t-test P-value	6.97e-18	1.71e-15	6.76e-18	7.81e-28	1.81e-18
Wilcoxon signed-rank test P-value	8.96e-20	1.40e-18	8.60e-20	7.77e-24	4.59e-21

**Table 3:** The average performance of each method on 134 ChIP-seq datasets.

Average AUC (%)	DREME	HOMER	XXmotif	motifRG	DECOD	WSMD
Training datasets	77.43	79.11	77.13	62.91	77.16	82.80
Test datasets	77.01	78.74	76.85	62.83	76.98	81.86
Average Fisher’s score (log10)	DREME	HOMER	XXmotif	motifRG	DECOD	WSMD
Training datasets	−37.42	−54.75	−57.81	−26.61	−59.77	−96.83
Test datasets	−18.50	−27.42	−27.77	−13.06	−29.37	−44.91
Average MHG score (log10)	DREME	HOMER	XXmotif	motifRG	DECOD	WSMD
Training datasets	−185.95	−187.62	−173.93	−112.67	−167.23	−219.87
Test datasets	−91.34	−92.82	−86.58	−55.83	−84.16	−108.08

For each tool, its average performance on both training and test datasets are presented.

Similarly, the Fisher’s Exact Test score (Fisher’s score) and Minimal Hyper-Geometric score (MHG score) (see Supplementary Section 3.1 & 3.2 for their rigorous mathematical definitions), which are respectively the learning objectives of DREME and HOMER, were used to quantify the relative enrichment of reported motifs in corresponding foreground datasets. Since Fisher’s Exact Test needs a pre-defined threshold to count the motif occurrence, following^35^, we set the threshold as the top 0.1% quantile of all site-level binding energies in the background sequences. Figure 2(b,c) presents the performance of four methods on Fisher’s score and MHG score. As it shows, WSMD performs orders of magnitude better than other methods on most of datasets. For example, the paired t-test and Wilcoxon signed-rank test between WSMD and DREME on Fisher’s score both returned very low P-values which highlight the advantages of WSMD in almost all datasets, even though DREME is specifically designed to optimize such a score (Table 2 rows 4–6). The average Fisher’s and MHG scores on all the 134 datasets for each algorithms are also reported in Table 3 (rows 4–9).

### Performance comparison on synthetic data

Our earlier studies on real ChIP-seq datasets have validated that the performance of WSMD is superior or competitive in comparison with DREME, HOMER, XXmotif, motifRG and DECOD. In this section, we tried to provide further insights on the advantages of our refinement and extension strategies based on artificially created test sequences. In contrast to the real datasets used in the previous sections, the constructive process of synthetic datasets explicitly defines the true binding positions, therefore the quality of elicited motif can be evaluated by directly assessing its accuracy for predicting binding sites on the nucleotide and binding-site level.

The setup for the simulation study is generally similar to those from previous works^8, 11, 33, 34^. More specifically, two sets of foreground and background datasets were firstly generated, each set consists of 2000 500 bp-long sequences that were sampled from a uniform distribution on DNA alphabets. Then, a signal PWM and a decoy PWM were respectively generated according to different settings of width and information content (IC). The signal PWM was only inserted into foreground sequences, and the decoy PWM was inserted into foreground and background sequences both. The parameters that varied in two sets of experiments are summarized in Table 4, including the width and IC of signal motifs and decoy motifs, and we generated 10 datasets for each set of parameters (more details about the synthetic datasets construction can be found in Supplementary Section 4). In terms of evaluation metrics, we also follow previous studies^13, 51^ and adopt the nucleotide-level correlation coefficient (nCC) and the average site-level performance (sASP) (see Supplementary Section 3.3 for their rigorous mathematical definitions). Additionally, in order to keep coherence between real and synthetic datasets, the performances of different methods on AUC, Fisher’s score and MHG score are also presented for synthetic datasets (see Supplementary Section 5).

**Table 4:** Parameters of implanted signal and decoy PWMs.

	Refinement	Extension
Signal width	8	18
Signal IC	2,4,6,8,10,12,14,16	6,8,10,12,14,16,18,20,22,24,26
Decoy width	8	18
Decoy IC	10	20
Total experiments	80	110

#### Performance comparison of refinement strategies

As mentioned before, in WSMD, the refinement of seed motifs is formulated as a unified learning problem(11), which could allow for simultaneous tuning of all motif parameters in continuous space. To confirm the advantage of this novel refinement strategy over the approximate schemes used by DREME, HOMER, XXmotif, motifRG and DECOD, we analyzed the performance of the six tested methods for motif refinement by using the same sets of seeds as input.

Concretely, to make the comparison fair enough, for each synthetic data, 5 seeds of length 8 were generated using our Seeding procedure, and fed into WSMD, HOMER and XXmotif for further refinement. Then, the best-performing motif reported by each method was used to evaluate its performance. Note that DREME, motifRG and DECOD does not allow optimization of a given motif, thus their performance are measured by running them on corresponding datasets with the width of motifs fixed at 8 and the maximum number of motifs fixed at 5.

**Figure 3:**
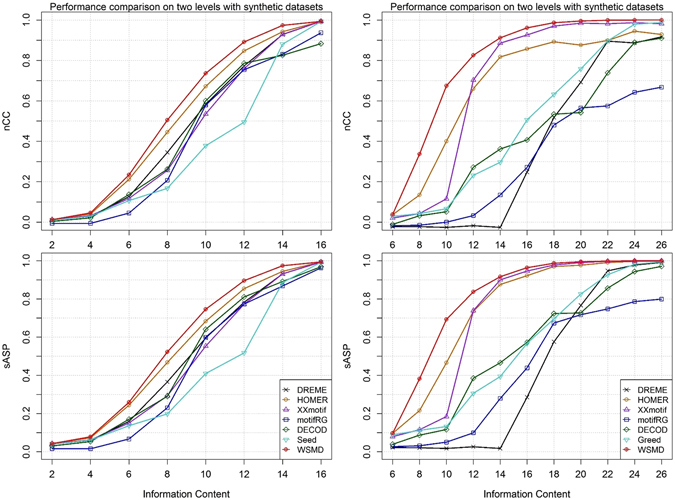
Performance comparison of different refinement and extension strategies. For each IC value, we show the average performance obtained by using each tools over 10 distinct synthetic datasets. (**a**) Performance comparison of different refinement strategies. (**b**) Performance comparison of different extension strategies.

Figure 3(a) summarizes the predictive performance of seeds and six DMD tools on datasets of increasing motif IC. The results show that WSMD almost always achieves the best predictive power, followed by HOMER and other tools. This illustrates the particular advantage of our refinement strategy over the ones used by HOMER and XXmotif. The performance comparison on AUC, Fisher’s score and MHG score also conform this conclusion (Figures S3–S5).

#### Performance comparison of extension strategies

Recall that in WSMD, the Extension stage considers all possible scenarios of extending seed motifs to the desired length. In addition, by using the same objective function, this stage is naturally integrated with the Refinement stage. This seems to be a more natural extension scheme compared with the one used by DREME, XXmotif and motifRG, which greedily extends the seed site-by-site until no improvement can be made, and the one used by HOMER and Seeder^38^ which simply appends (l−k)/2 positions at both sides symmetrically. In order to validate the benefits of our extension strategy, we conducted extensive experiments on more challenging synthetic datasets described in Table 3. More specifically, WSMD, HOMER and XXmotif were initialized with 5 pre-generated seeds of length 6 and required to recognize a motif of length 18. Besides these three methods, a variant of WSMD, referred to as Greed, was implemented to simulate the greedy extension strategy used by DREME and motifRG. Greed uses the same learning procedure as WSMD, with the exception that the refined motif is extended greedily until the desire length is achieved. Figure 3(b) summarizes the prediction performance of each tool on extension experiments by nCC and sASP. As it shows, WSMD has a significant advantage over any other tested algorithm, which confirms the soundness of formulating extension as a unified optimization task. On the other hand, both DREME and motifRG perform poorly on these datasets, which is expected as they only greedily search for motifs in a discrete space.

#### Running time comparison

**Figure 4:**
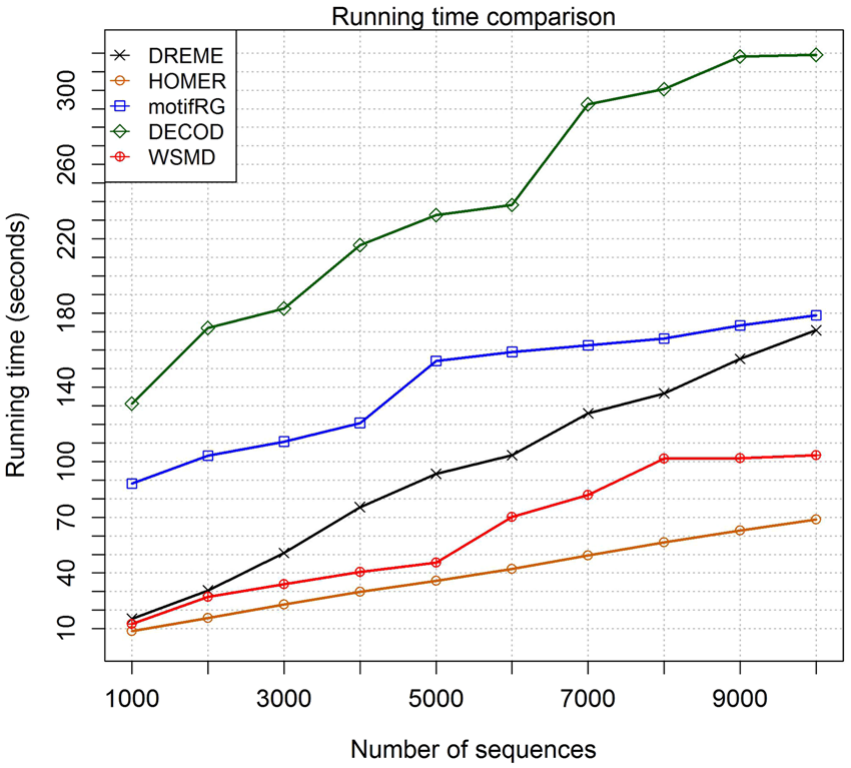
Comparison of running time (seconds) for DREME, HOMER, motifRG, DECOD and WSMD.

WSMD was implemented with R language. We compared the running time of the six DMD tools on datasets of increasing size. All algorithms were performed on a 3.4 GHz 4-core computer running 64bit-Linux. For each setting of dataset size, the experiment were repeated 10 times and the averaged results were reported. Figure 4 plots the average running time in seconds against the number of sequences. The performance of XXmotif was not presented in the Fig. 4 because its running time is significantly longer, for example it spent 4.413 minutes when dealing with dataset containing 1000 sequences and the running time exceeded half an hour when increasing the dataset size to 10000 (Supplementary Table S2). The results show that WSMD is faster than DREME, XXmotif, motifRG and DECOD, while it is still slower than HOMER. However, it has to be emphasized that the search space of HOMER is much smaller and could significantly sacrifice accuracy, as is indicated by previous experiments.

## Discussion

In this article, we pointed out the inherent similarities between DMD and OD and thereby proposed a novel method for identifying motifs from ChIP-seq datasets. The core idea of our approach is to learn the optimal motifs by maximizing classification accuracy, which is one of the most popular metrics in pattern classification. Through rigorous mathematical deduction, we proved that the CA-based DMD problem is essentially equivalent to latent support vector machine (LSVM), which is a widely studied formulation of WSL. WSMD outperforms other popular motif finding tools by: (i) Searching for motifs in a continuous space, which could greatly reduce the information loss of model representation; (ii) Formulating DMD problem as an integrated optimization task in which PWM could be refined directly. When tested on real ChIP-seq and synthetic datasets, we showed that the motifs found by WSMD have an excellent performance based on various evaluation criteria. In further experiments on synthetic datasets, where the well-defined ‘correct’ outcomes are known, WSMD outperforms all benchmarked methods when searching for complicated motifs. Meanwhile, by incorporating ideas from several existing OD literatures, WSMD could also archive competitive speed.

Here, we primarily focus on discussing TFBS motif learning alone. However, it is important to emphasize that the mechanisms by which TFs select their functional binding sites in a cellular environment are highly complex and do not rely purely on recognitions of motifs^48, 52^. In fact, TFs bind only a small fraction of regions that match their corresponding motifs in any given biological condition^48, 53^, which means that motif learning alone cannot accurately predict TF binding *in vivo* ^43^. For instance, TF binding could be significantly altered by many external factors, such as the DNA shape^54^, protein concentration^55^, nucleosomes^56, 57^, chromatin accessibility^58^, cofactors^59, 60^, and pioneer TFs^57^, etc. Previous researches have also demonstrated that modeling of TF-DNA interactions can benefit greatly from incorporation of these non-sequence features^58, 61–63^.

In addition, even if one chooses to only model the sequence specificities of TFs, direct application of motif elicitation tools is still not the ideal choice and does not perform well in practice^43, 64^. This is because the sequence information recognized by a TF is also not limited to the core-binding motif^64^. For example, the lower-order sequence composition (e.g., GC content) of DNA regions that most TFs bind to is often different from that of the rest of the genome^65–67^. In addition, clustered weak binding motifs are often found in the local sequence environment around the core site^64, 68^, which is hypothesized to reduce genetic perturbations and help the TFs to reduce the search space of binding sites^64^. Due to these issues, top-performing machine learning methods for sequence-based modeling^43, 69–71^ are generally SVMs (e.g., gkmSVM^72^ and SeqGL^43^) or neural nets (e.g., DeepBind^69^ and Basset^70^) trained using a large set of features that collectively capture the complex properties of bound DNA sequences.

Meanwhile, although motif learning has the limitations mentioned above, it still plays indispensable roles in TF binding study. Firstly, motif information remains to be an integral part of binding models that could incorporate multilayered genomic datasets^61–63^. While dedicated motif databases such as JASPAR^46^ and TRANSFAC^44^ exist, they are far from complete and a large number of motifs still need to be characterized, such as the motifs of heterodimers. Thus novel computational and experimental technologies are in need to bridge this gap; Secondly, although recent sequence models such as SeqGL and DeepBind show their potential for modeling the overall binding affinity of TFs, they are “black boxes” in nature and difficult to interpret. As a result, *de novo* motif discovery tools and/or motif databases are typically used in the end to analyze outputs of these advanced models^43, 58, 70^.

Finally, in spite of its good performance, WSMD still leaves some room for improvement in our previous analysis: (i) The present implementation of WSMD only allows for searching PWMs on ChIP-seq datasets. It is worth studying that whether WSMD could be extended to RNA or protein sequence analysis, as well as to high-order motif models. (ii) Although WSMD can outperform other methods by utilizing a simple coordinate-descent-style learning strategy, its classification accuracy based discriminative object function is still nonconvex and could lead to local minima. There is potential to further improve the performance of WSMD by adapting some more sophisticated strategies discussed in the WSL literatures^23, 73^. Although the above-mentioned future directions are conceptually feasible, they also inevitably lead to more complex learning problems that are computationally expensive in practice. Therefore, we will focus on further exploring the possibilities of applying these ideas without sacrificing scalability.

## Electronic supplementary material

Supplementary Material
