# Supplementary material for: WSMD: weakly-supervised motif discovery in transcription factor ChIP-seq data
Source: Sci Rep. 2017 Jun 12;7:3217. doi: 10.1038/s41598-017-03554-7 (PMC5468353; doi:10.1038/s41598-017-03554-7)
Supplement: Supplementary file 1 — Supplementary Material [file 41598_2017_3554_MOESM1_ESM.pdf]

WSMD: weakly-supervised motif discovery in transcription factor ChIP-seq data

——Supplementary Material

Hongbo Zhang[1*], Lin Zhu[1*] and De-Shuang Huang[1]

1. Detail description of WSMD

1.1. Overview of WSMD

Inherited and adapted from traditional DMD framework, WSMD consists of five steps (Fig. S1.): Preprocessing, Seeding, Refinement, Extension and Masking. Firstly, in "Preprocess" step, the input foreground and background sequences are transformed as positive and negative bags respectively. Then, in the "Seeding" step, some motif proposals are obtained based on the discrimination scores of enumerated 6-mers. Then, these seeds are fed into "Refinement" step for further optimization with an iterative strategy. "Extension", following "Refinement", is applied to extend the refined motif to a desired length. Finally, the "Masking" step, masks all of the occurrences for the reported motif. After completing masking and outputting results, the four stages in the right pane will resume to find other motifs. Next we provide a high-level description of the above five steps.

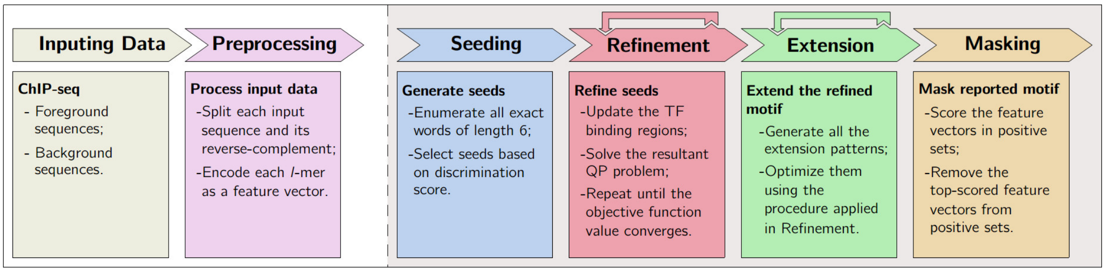

Fig. S1. The overview of WSMD.

1.2. Preprocessing

In the scenario of DMD, the input sequence is labelled as foreground or background to denote whether it contains a motif occurrence. Similar as image segmentation in OD, WSMD needs to split the input sequences into short regions. An outline of sequence splitter is showed in Fig. S2. Specifically, we scan a sequence from left to right with an *l*-length sliding window, where *l* is the desired motif length. Then, the regions belonged to the same sequence are group into a same bag. Since the foreground sequence contains a motif occurrence at least, it can be formulated as a positive bag of *l*-mers. Similarly, the background sequences are formulated as negative bags. Additionally, for each input sequence, the TFBS can be located either on up or down strand, therefore, we also process its reverse-complement

1 Institute of Machine Learning and Systems Biology, College of Electronics and Information Engineering, Tongji University, Shanghai, 201804, P.R. China. *These authors contributed equally to this work. Correspondence and requests for materials should be addressed to De-Shuang Huang (email: dshuang@tongji.edu.cn)

with the same procedure. At last, we encode each l-mer as a $4l$-length binary feature vector by transforming each nucleotide into a 4-dimensional vector using "one-hot encoding":

$$\begin{cases} \text{"A"} = \{1,0,0,0\}, \ \text{"C"} = \{0,1,0,0\}, \\ \text{"G"} = \{0,0,1,0\}, \ \text{"T"} = \{0,0,0,1\}. \end{cases} \tag{1}$$

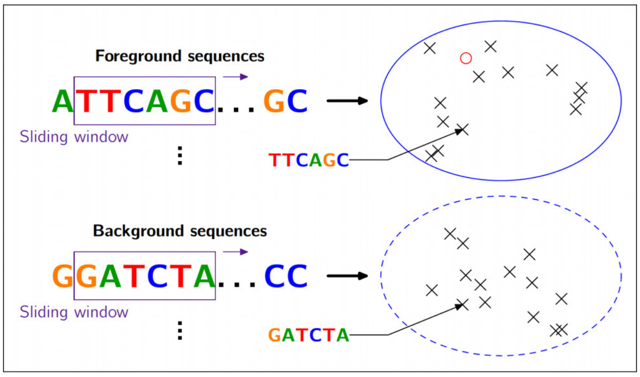

Fig. S2. The outline of sequence splitter. The circle with solid line represents a positive bag and that with dashed line represents a negative bag. Each bag consists of all the l-mers from one sequence, the red circle represents a positive instance and the cross represents a negative one.

1.3. Seeding

Given foreground dataset $\boldsymbol{F}$, background dataset $\boldsymbol{B}$ and an l-length seed $\mathbf{s}$, we first transform $\mathbf{s}$ into a standard PWM $\mathbf{P} \in \mathbb{R}_{4\times l}$ using "one-hot encoding" given in equation(1). Following previous works[1-4], we evaluate the binding energy of a DNA sequence whose length is over l, $\mathbf{x} = (x_1 \dots x_m), m > l$, with its maximal site-level binding energy. Formally, we have

$$\mathrm{E}(\mathbf{P},\mathbf{x}) = \max\left(\sum_{i=j}^{j+l-1} \log\left(\mathbf{P}_{(x_i,i)}\right), j \in [1, m-l+1] \right) . \tag{2}$$

For $\boldsymbol{F}$ and $\boldsymbol{B}$, the binding energy of each sequence with seed $\mathbf{s}$ can be calculated using(2) and defined as follows:

$$\begin{cases} \mathbf{f} = \{f_i\}, \text{where } f_i = \mathrm{E}(\mathbf{P},\mathbf{x}_i), \mathbf{x}_i \in \boldsymbol{F}, \\ \mathbf{b} = \{b_i\}, \text{where } b_i = \mathrm{E}(\mathbf{P},\mathbf{x}_i), \mathbf{x}_i \in \boldsymbol{B}. \end{cases} \tag{3}$$

We evaluate the "discrimination score" of seed $\mathbf{s}$ by calculating the probability $p(f > b)$, where f and b are random variables selected from $\mathbf{f}$ and $\mathbf{b}$ respectively. Formally we have

$$\mathrm{D}(\mathbf{s},\boldsymbol{F},\boldsymbol{B}) = \frac{\sum_{i=1}^{|\boldsymbol{F}|}\sum_{j=1}^{|\boldsymbol{B}|}\left(\mathrm{sgn}(f_i > b_j) + \frac{1}{2}\mathrm{sgn}(f_i = b_j) \right)}{|\boldsymbol{F}||\boldsymbol{B}|} , \tag{4}$$

where $\mathrm{sgn}(\cdot)$ is an indicator function which return 1 if the argument is true and 0 otherwise.

It is worth noting that there is no need to apply one-hot encoding in the practical implementation of this procedure, since all the seeds and sequences consist of exact characters over the DNA alphabet. And in this particular case, the binding energy of $\mathbf{s}$ with an equal length sequence $\mathbf{y}$ can be equivalent

to the number of sites where they have identical characters. Formally we have

$$\mathrm{E}(\mathbf{s},\mathbf{x})=\sum_{i=1}^{|\mathbf{s}|}\operatorname{sgn}(s_i=y_i)=|\mathbf{s}|-\mathrm{H}(\mathbf{s},\mathbf{y}), \tag{5}$$

where $\mathrm{H}(\mathbf{s},\mathbf{y})$ return the hamming distance between sequence **s** and **y**. Using this notation, we can rewrite(5) as

$$\mathrm{E}(\mathbf{s},\mathbf{x})=\max_{\mathbf{y}\in\boldsymbol{X}}\left(\sum_{i=1}^{l}\operatorname{sgn}(s_i=y_i)\right) \tag{6}$$

$$=\max_{\mathbf{y}\in\boldsymbol{X}}\left(|\mathbf{s}|-\mathrm{H}(\mathbf{s},\mathbf{y})\right), \tag{7}$$

Where $\boldsymbol{X}$ consists of all the possible l-mers of **x**. Consider(7) and remove its outside brackets, then we get

$$\mathrm{E}(\mathbf{s},\mathbf{x})=|\mathbf{s}|-\min_{\mathbf{y}\in\boldsymbol{X}}\left(\mathrm{H}(\mathbf{s},\mathbf{y})\right). \tag{8}$$

It denotes that the bound energy of **s** with **x** is directly related to the Hamming distance (HD) between **s** and its best matching l-mer in $\boldsymbol{X}$ (named the substring minimal distance—SMD).

In this way, we transformed the original problem into a string matching task: finding the best matching l-mer in each input sequence for a given l-length seed. We developed an approximate solution based on the experimental observation that for large-scale ChIP-seq datasets, the best matching l-mer of a seed in most sequences is either the seed itself or very similar seeds which differ with the seed in only one site. This is mainly due to following fact: as in general DMD methods, the length of the seed is typically short to achieve high speed (default to 6 in most situations), yet current experimental techniques for determining TF specificity could only locate the binding site within a region of the genome that is significantly longer (hundreds to thousands). Therefore, it becomes highly likely for a seed to find a very similar matching even in background sequences.

Suppose that given an l-length seed **s** attached with seed set $\boldsymbol{S}$ which contains all the l-length seeds that have hamming distance of 1 with **s**. For the input foreground sequences set $\boldsymbol{F}$ and background sequences set $\boldsymbol{B}$, the number of sequences which contain **s** is counted and represented as f_0 and b_0 respectively; and the number of other foreground and background sequences which contain any seed belong to $\boldsymbol{S}$, represented as f_1 and b_1 respectively. Then the approximate of(4) can be written as follow

$$\overline{\mathrm{D}}(\mathbf{s},\boldsymbol{F},\boldsymbol{B})=\frac{\left(f_0\left(b_1+b\right)+f_1 b+\frac{1}{2}\left(f_0 b_0+f_1 b_1+fb\right)\right)}{|\boldsymbol{F}||\boldsymbol{B}|}, \tag{9}$$

where $f=|\boldsymbol{F}|-f_0-f_1$ and $b=|\boldsymbol{B}|-b_0-b_1$. Based on this approximate solution, the procedure of "Seeding" stage is detailed in Algorithm S1. Note that, n is a user-defined variable to set the number of candidate motifs. Additionally, we count the occurrence information by scanning the input data sequence-by-sequence rather than seed-by-seed. This is motivated by the implementation technique that the approximate discriminability of all the seeds can be evaluated by scanning the sequences for just once with the help of SMD *Index*. Here, we calculate SMD *Index* as follow, we first enumerate all the possible *6*-mers, and store them in a sequence vector set $\boldsymbol{S}=(\mathbf{s}_i),\mathbf{s}_i\in\mathbb{R}_l,|\boldsymbol{S}|=4^6$. Then we

construct SMD *Index* **H** defined as:

$$\mathbf{H} = \left(h_{ij}\right) \in \mathbb{R}_{4^6 \times 18}, \mathbf{H}_{(i,*)} = \left\{x \mid \mathrm{H}\left(\mathbf{s}_i, \mathbf{s}_x\right) = 1, \mathbf{s}_x \in \boldsymbol{S}\right\}. \tag{10}$$

Exactly, for $\mathbf{s}_i$, the row vector $\mathbf{H}_{(i,*)}$ contains the index of seeds which have hamming distance of one with $\mathbf{s}_i$. It is worth noting that neither $\boldsymbol{S}$ nor $\mathbf{H}$ depends on the input dataset, and therefore can be pre-computed and stored.

Algorithm S1 Seeding stage

Input: the number of seeds *n*, foreground dataset ***F***, background dataset ***B***, all the possible 6-mers ***S*** SMD *Index* **H**.

Output: A set of candidate motifs ***C***.

```
while f in F
    count the occurrence of S in f;
    calculate f_0 and f_1 for each seed by querying H;
end while
while b in B
    count the occurrence of S in b;
    calculate b_0 and b_1 for each seed by querying H;
end while
while s in S
    calculate D̄(s, F, B) using(9);
end while
sort seeds in descending order of their D̄ ;
return C ← {x ∈ S | D̄(x, F, B) ≥ D̄(y, F, B), ∀y ∈ S \ C}, |C| = n .
```

1.4. Refinement

In the refinement step, WSMD takes the highest scored *k*-mers from the seeding step and transforms them into PWMs, then optimizes them to obtain refined PWMs by solving the following task:

$$\begin{aligned} &\min_{\mathbf{w},\xi,b} \|\mathbf{w}\|_2^2 + \frac{C}{|\boldsymbol{F}| + |\boldsymbol{B}|} \sum \xi_i, \\ &\text{s.t. } y_{\mathbf{s}} \left(\max_{\mathbf{s}^{\text{sub}} \in \boldsymbol{S}} \left(\mathbf{w}^T \mathbf{x}^{\text{sub}} \right) - b \right) + \xi_i \geq 1,\ \xi_i \geq 0, \forall \mathbf{s} \in \boldsymbol{F} \cup \boldsymbol{B}. \end{aligned} \tag{11}$$

Here, we can still solve(11) efficiently by using the coordinate-descent-style LSVM optimization strategy proposed in[5]. The basic idea is to exploit the fact that if the latent variables that mark the bound regions of each input sequences are given, the problem(11) reduces to a convex quadratic programming (QP) which can be solved exactly. We score sequence $\mathbf{s}_i \in \boldsymbol{F} \cup \boldsymbol{B}$ with its maximal site-level binding energy, which is equivalent to selecting a single possible latent value $\mathbf{x}_i$ for $\mathbf{s}_i$, then we obtain a set of labeled examples $\boldsymbol{G} = \left\{\left(\mathbf{x}_1, y_1\right), \ldots, \left(\mathbf{x}_m, y_m\right)\right\}$, where $y_i \in \{1, -1\}$ and $y_i = 1$ if $\mathbf{s}_i \in \boldsymbol{F}$ and $y_i = -1$ otherwise. Then the problem(11) reduces to a linear SVM defined as follows

$$\min_{\mathbf{w},\xi,b} \|\mathbf{w}\|_2^2 + \frac{c}{|\boldsymbol{F}|+|\boldsymbol{B}|}\sum \xi_i,$$
$$\text{s.t. } y_i\left(\mathbf{w}^{\mathrm{T}}\mathbf{x}_i\right)+\xi_i \geq 1, \xi_i \geq 0, i \in [1,m]. \tag{12}$$

(12) can be solved efficiently using off-the-shelf software such as Mosek and CPLEX. Algorithm S2 summarizes pseudo-code descriptions for refinement step. More specifically, the PWMs are improved iteratively with two alternating steps: **Update-step**: Update the bound regions for both foreground and background sequences using the current PWM; **QP-step**: Solve the associated QP problem to update PWM. This procedure is repeated until the objective function value converges.

When learning a motif for DMD we often have a very large number of background sequences. Here, it is infeasible to consider all of them simultaneously since the enormous amount of background sequences may lead to huge computational costs. Likewise, many OD methods too, are faced with this problem. To overcome such difficulty, they choose to learn models using only "hard negative" instances instead of using all of negative examples[6,7]. For the motif learning setting considered here, such a strategy means that during the Update-step, we maintain a "hard negative" cache for background sequences, and update it with a two-stage strategy(Algorithm S3): **Growing the cache** by adding the background regions with the highest site-level binding energy using the current PWM. **Shrinking the cache** by removing the background regions with relatively low site-level binding energy to prevent it from exceeding a user-defined size limit.

Algorithm S2 Refinement step

Input: Seed **s**, sequence set ***F*** and ***B***

Output: The optimized PWM **P**.

```
initialize w with s
repeat
    update G = {(x_1, y_1), ..., (x_m, y_m)} with Algorithm 2;
    solve the QP in (12) to obtain w, b with labeled examples G;
until the objective function value converges;
transform and normalize w to get the PWM P;
return P.
```

1.5. Extension

Due to the rapidly increasing of search space and computational cost with the motif width, almost all the de novo MD methods start with a relatively short width, and then extend it to a suitable width. The common extension strategy is extending one by one site at either sides of current motif. Essentially, it is a greedy algorithm based on the assumption that a segment of the optimal motif must also be the optimal one. In actually, this is an unreasonable strategy, since its assumption may be invalid in some situation, and the deviation may ascend as the site by site extension process. Here, we describe another extension strategy. Suppose that extending a k-length motif $\mathbf{P}^k$ to width $l>k$. Firstly, we add uniform weights at x positions upstream and l-k-x positions downstream of the motif respectively, where x varies between 0 and l-k. Such a protocol yields l-k+1 initial PWMs of length l, $\mathbf{P} = \left\{\mathbf{P}_i^l, i \in [1, l-k+1]\right\}$, which are then again optimized using the same procedure adopted in

Refinement, and the one that achieves the minimal objective function value is reported as the final motif.

Algorithm S3 Update $\boldsymbol{G}$

Input: $\mathbf{w}$, sequence set $\boldsymbol{F}$ and $\boldsymbol{B}$, hard negative cache size n.

Output: Labeled examples $\boldsymbol{G} = \{(\mathbf{x}_1, y_1), \ldots, (\mathbf{x}_m, y_m)\}, y_i \in \{-1, 1\}$.

$\boldsymbol{G}_f \leftarrow \left\{ (\mathbf{x}_i, 1) \mid \mathbf{x}_i = \arg\max_{\mathbf{x}^{\text{sub}}} \left(\mathbf{w}^{\mathrm{T}} \mathbf{x}^{\text{sub}}, \mathbf{s}^{\text{sub}} \in \boldsymbol{F} \right) \right\}$;

$\boldsymbol{G}_b \leftarrow \boldsymbol{G}_b \cup \left\{ (\mathbf{x}_i, -1) \mid \mathbf{x}_i = \arg\max_{\mathbf{x}^{\text{sub}}} \left(\mathbf{w}^{\mathrm{T}} \mathbf{x}^{\text{sub}}, \mathbf{s}^{\text{sub}} \in \boldsymbol{B} \right) \right\}$;

if $|\boldsymbol{G}_b| > n$

$\boldsymbol{T} \leftarrow \left\{ (x_i, -1) \in \boldsymbol{G}_b \mid \mathbf{w}^{\mathrm{T}} \mathbf{x}_i \geq \mathbf{w}^{\mathrm{T}} \mathbf{x}_j, \forall (x_j, -1) \in \boldsymbol{G}_b \setminus \boldsymbol{T} \right\}, |\boldsymbol{T}| = n$;

    $\boldsymbol{G}_b \leftarrow \boldsymbol{T}$;

return $\boldsymbol{G} \leftarrow \boldsymbol{G}_f \cup \boldsymbol{G}_b$.

1.6. Masking

Due to the cooperative binding of TFs, it is highly likely that more than one motif is relevant to the ChIP-seq dataset being analyzed, which requests motif finders that can extract multiple non-redundant motifs from a set of input sequences. A common adopted strategy to fulfill such a requirement is to mask the "most potential" binding regions of foreground sequences for the reported motif, and then repeat the search procedure for other motifs. In WSMD, this can be done by scoring the feature vectors in positive bags first, and then removing the top-scored feature vectors from the positive set.

2. The complete list of real ChIP-seq datasets

We collected 134 ENCODE datasets from two groups, Haib_Tfbs (available from http://hgdownload.cse.ucsc.edu/goldenpath/hg19/encodeDCC/wgEncodeHaibTfbs/) and Sydh_Tfbs (available from http://hgdownload.cse.ucsc.edu/goldenpath/hg19/encodeDCC/wgEncodeSydhTfbs/), their file names and the dataset IDs used in our work are list below:

Table S1. List of 134 real ChIP-seq datasets

Dataset ID	Dataset file names
1	wgEncodeHaibTfbsA549Usf1Pcr1xDex100nmPkRep1.broadPeak.gz
2	wgEncodeHaibTfbsA549Usf1Pcr1xEtoh02PkRep1.broadPeak.gz
3	wgEncodeHaibTfbsEcc1EralphaaV0416102Gen1hPkRep1.broadPeak.gz
4	wgEncodeHaibTfbsGm12878Atf3Pcr1xPkRep1.broadPeak.gz
5	wgEncodeHaibTfbsGm12878Ebfsc137065Pcr1xPkRep1.broadPeak.gz
6	wgEncodeHaibTfbsGm12878Egr1V0416101PkRep1.broadPeak.gz
7	wgEncodeHaibTfbsGm12878Elf1sc631V0416101PkRep1.broadPeak.gz
8	wgEncodeHaibTfbsGm12878Ets1Pcr1xPkRep1.broadPeak.gz
9	wgEncodeHaibTfbsGm12878GabpPcr2xPkRep1.broadPeak.gz

10	wgEncodeHaibTfbsGm12878Irf4sc6059Pcr1xPkRep1.broadPeak.gz
11	wgEncodeHaibTfbsGm12878Mef2aPcr1xPkRep1.broadPeak.gz
12	wgEncodeHaibTfbsGm12878Mef2csc13268V0416101PkRep1.broadPeak.gz
13	wgEncodeHaibTfbsGm12878NrsfPcr2xPkRep1.broadPeak.gz
14	wgEncodeHaibTfbsGm12878Pax5c20Pcr1xPkRep1.broadPeak.gz
15	wgEncodeHaibTfbsGm12878Pax5n19Pcr1xPkRep1.broadPeak.gz
16	wgEncodeHaibTfbsGm12878Pbx3Pcr1xPkRep1.broadPeak.gz
17	wgEncodeHaibTfbsGm12878Pou2f2Pcr1xPkRep1.broadPeak.gz
18	wgEncodeHaibTfbsGm12878Pu1Pcr1xPkRep1.broadPeak.gz
19	wgEncodeHaibTfbsGm12878RxraPcr1xPkRep1.broadPeak.gz
20	wgEncodeHaibTfbsGm12878Six5Pcr1xPkRep1.broadPeak.gz
21	wgEncodeHaibTfbsGm12878Sp1Pcr1xPkRep1.broadPeak.gz
22	wgEncodeHaibTfbsGm12878SrfPcr2xPkRep1.broadPeak.gz
23	wgEncodeHaibTfbsGm12878SrfV0416101PkRep1.broadPeak.gz
24	wgEncodeHaibTfbsGm12878Tcf12Pcr1xPkRep1.broadPeak.gz
25	wgEncodeHaibTfbsGm12878Usf1Pcr2xPkRep1.broadPeak.gz
26	wgEncodeHaibTfbsGm12878Yy1sc281Pcr1xPkRep1.broadPeak.gz
27	wgEncodeHaibTfbsGm12878Zeb1sc25388V0416102PkRep1.broadPeak.gz
28	wgEncodeHaibTfbsGm12891Pax5c20V0416101PkRep1.broadPeak.gz
29	wgEncodeHaibTfbsGm12891Pou2f2Pcr1xPkRep1.broadPeak.gz
30	wgEncodeHaibTfbsGm12891Pu1Pcr1xPkRep1.broadPeak.gz
31	wgEncodeHaibTfbsGm12891Yy1sc281V0416101PkRep1.broadPeak.gz
32	wgEncodeHaibTfbsGm12892Pax5c20V0416101PkRep1.broadPeak.gz
33	wgEncodeHaibTfbsGm12892Yy1V0416101PkRep1.broadPeak.gz
34	wgEncodeHaibTfbsH1hescAtf3V0416102PkRep1.broadPeak.gz
35	wgEncodeHaibTfbsH1hescEgr1V0416102PkRep1.broadPeak.gz
36	wgEncodeHaibTfbsH1hescFosl1sc183V0416102PkRep1.broadPeak.gz
37	wgEncodeHaibTfbsH1hescGabpPcr1xPkRep1.broadPeak.gz
38	wgEncodeHaibTfbsH1hescJundV0416102PkRep1.broadPeak.gz
39	wgEncodeHaibTfbsH1hescNrsfV0416102PkRep1.broadPeak.gz
40	wgEncodeHaibTfbsH1hescPou5f1sc9081V0416102PkRep1.broadPeak.gz
41	wgEncodeHaibTfbsH1hescRxraV0416102PkRep1.broadPeak.gz
42	wgEncodeHaibTfbsH1hescSix5Pcr1xPkRep1.broadPeak.gz
43	wgEncodeHaibTfbsH1hescSp1Pcr1xPkRep1.broadPeak.gz
44	wgEncodeHaibTfbsH1hescSrfPcr1xPkRep1.broadPeak.gz
45	wgEncodeHaibTfbsH1hescTcf12Pcr1xPkRep1.broadPeak.gz
46	wgEncodeHaibTfbsH1hescUsf1Pcr1xPkRep1.broadPeak.gz
47	wgEncodeHaibTfbsH1hescYy1sc281V0416102PkRep1.broadPeak.gz
48	wgEncodeHaibTfbsHelas3GabpPcr1xPkRep1.broadPeak.gz
49	wgEncodeHaibTfbsHelas3NrsfPcr1xPkRep1.broadPeak.gz
50	wgEncodeHaibTfbsHepg2Atf3V0416101PkRep1.broadPeak.gz
51	wgEncodeHaibTfbsHepg2Bhlhe40V0416101PkRep1.broadPeak.gz
52	wgEncodeHaibTfbsHepg2Elf1sc631V0416101PkRep1.broadPeak.gz

53	wgEncodeHaibTfbsHepg2Fosl2V0416101PkRep1.broadPeak.gz
54	wgEncodeHaibTfbsHepg2Foxa1sc101058V0416101PkRep1.broadPeak.gz
55	wgEncodeHaibTfbsHepg2Foxa1sc6553V0416101PkRep1.broadPeak.gz
56	wgEncodeHaibTfbsHepg2Foxa2sc6554V0416101PkRep1.broadPeak.gz
57	wgEncodeHaibTfbsHepg2GabpPcr2xPkRep1.broadPeak.gz
58	wgEncodeHaibTfbsHepg2Hnf4asc8987V0416101PkRep1.broadPeak.gz
59	wgEncodeHaibTfbsHepg2Hnf4gsc6558V0416101PkRep1.broadPeak.gz
60	wgEncodeHaibTfbsHepg2JundPcr1xPkRep1.broadPeak.gz
61	wgEncodeHaibTfbsHepg2NrsfPcr2xPkRep1.broadPeak.gz
62	wgEncodeHaibTfbsHepg2RxraPcr1xPkRep1.broadPeak.gz
63	wgEncodeHaibTfbsHepg2Sp1Pcr1xPkRep1.broadPeak.gz
64	wgEncodeHaibTfbsHepg2SrfV0416101PkRep1.broadPeak.gz
65	wgEncodeHaibTfbsHepg2Tcf12Pcr1xPkRep1.broadPeak.gz
66	wgEncodeHaibTfbsHepg2Usf1Pcr1xPkRep1.broadPeak.gz
67	wgEncodeHaibTfbsHepg2Yy1sc281V0416101PkRep1.broadPeak.gz
68	wgEncodeHaibTfbsK562Atf3V0416101PkRep1.broadPeak.gz
69	wgEncodeHaibTfbsK562E2f6sc22823V0416102PkRep1.broadPeak.gz
70	wgEncodeHaibTfbsK562Egr1V0416101PkRep1.broadPeak.gz
71	wgEncodeHaibTfbsK562Elf1sc631V0416102PkRep1.broadPeak.gz
72	wgEncodeHaibTfbsK562Ets1V0416101PkRep1.broadPeak.gz
73	wgEncodeHaibTfbsK562Fosl1sc183V0416101PkRep1.broadPeak.gz
74	wgEncodeHaibTfbsK562GabpV0416101PkRep1.broadPeak.gz
75	wgEncodeHaibTfbsK562Gata2sc267Pcr1xPkRep1.broadPeak.gz
76	wgEncodeHaibTfbsK562MaxV0416102PkRep1.broadPeak.gz
77	wgEncodeHaibTfbsK562Mef2aV0416101PkRep1.broadPeak.gz
78	wgEncodeHaibTfbsK562NrsfV0416102PkRep1.broadPeak.gz
79	wgEncodeHaibTfbsK562Pu1Pcr1xPkRep1.broadPeak.gz
80	wgEncodeHaibTfbsK562Six5Pcr1xPkRep1.broadPeak.gz
81	wgEncodeHaibTfbsK562Six5V0416101PkRep1.broadPeak.gz
82	wgEncodeHaibTfbsK562Sp1Pcr1xPkRep1.broadPeak.gz
83	wgEncodeHaibTfbsK562Sp2sc643V0416102PkRep1.broadPeak.gz
84	wgEncodeHaibTfbsK562SrfV0416101PkRep1.broadPeak.gz
85	wgEncodeHaibTfbsK562Usf1V0416101PkRep1.broadPeak.gz
86	wgEncodeHaibTfbsK562Yy1V0416101PkRep1.broadPeak.gz
87	wgEncodeHaibTfbsK562Yy1V0416102PkRep1.broadPeak.gz
88	wgEncodeHaibTfbsPanc1NrsfPcr2xPkRep1.broadPeak.gz
89	wgEncodeHaibTfbsPfsk1NrsfPcr2xPkRep1.broadPeak.gz
90	wgEncodeHaibTfbsSknshraUsf1sc8983V0416102PkRep1.broadPeak.gz
91	wgEncodeHaibTfbsSknshraYy1sc281V0416102PkRep1.broadPeak.gz
92	wgEncodeHaibTfbsT47dEralphaaPcr2xGen1hPkRep1.broadPeak.gz
93	wgEncodeHaibTfbsU87NrsfPcr2xPkRep1.broadPeak.gz
94	wgEncodeSydhTfbsGm12878Brca1a300IggmusPk.narrowPeak.gz
95	wgEncodeSydhTfbsGm12878Ebf1sc137065StdPk.narrowPeak.gz

96	wgEncodeSydhTfbsGm12878Nfe2sc22827StdPk.narrowPeak.gz
97	wgEncodeSydhTfbsGm12878Stat1StdPk.narrowPeak.gz
98	wgEncodeSydhTfbsGm12878Stat3IggmusPk.narrowPeak.gz
99	wgEncodeSydhTfbsGm12878Usf2IggmusPk.narrowPeak.gz
100	wgEncodeSydhTfbsGm12878Znf143166181apStdPk.narrowPeak.gz
101	wgEncodeSydhTfbsH1hescCjunIggrabPk.narrowPeak.gz
102	wgEncodeSydhTfbsH1hescMaxUcdPk.narrowPeak.gz
103	wgEncodeSydhTfbsH1hescRfx5200401194IggrabPk.narrowPeak.gz
104	wgEncodeSydhTfbsH1hescUsf2IggrabPk.narrowPeak.gz
105	wgEncodeSydhTfbsHelas3Brca1a300IggrabPk.narrowPeak.gz
106	wgEncodeSydhTfbsHelas3CebpbIggrabPk.narrowPeak.gz
107	wgEncodeSydhTfbsHelas3Elk4UcdPk.narrowPeak.gz
108	wgEncodeSydhTfbsHelas3Irf3IggrabPk.narrowPeak.gz
109	wgEncodeSydhTfbsHelas3Stat3IggrabPk.narrowPeak.gz
110	wgEncodeSydhTfbsHelas3Usf2IggmusPk.narrowPeak.gz
111	wgEncodeSydhTfbsHepg2CebpbIggrabPk.narrowPeak.gz
112	wgEncodeSydhTfbsHepg2CjunIggrabPk.narrowPeak.gz
113	wgEncodeSydhTfbsHepg2Irf3IggrabPk.narrowPeak.gz
114	wgEncodeSydhTfbsHepg2JundIggrabPk.narrowPeak.gz
115	wgEncodeSydhTfbsHepg2Maffm8194IggrabPk.narrowPeak.gz
116	wgEncodeSydhTfbsHepg2Mafkab50322IggrabPk.narrowPeak.gz
117	wgEncodeSydhTfbsHepg2Mafksc477IggrabPk.narrowPeak.gz
118	wgEncodeSydhTfbsHepg2Usf2IggrabPk.narrowPeak.gz
119	wgEncodeSydhTfbsHuvecCfosUcdPk.narrowPeak.gz
120	wgEncodeSydhTfbsHuvecGata2UcdPk.narrowPeak.gz
121	wgEncodeSydhTfbsK562Bhlhe40nb100IggrabPk.narrowPeak.gz
122	wgEncodeSydhTfbsK562CebpbIggrabPk.narrowPeak.gz
123	wgEncodeSydhTfbsK562CmycIfng30StdPk.narrowPeak.gz
124	wgEncodeSydhTfbsK562Irf1Ifna30StdPk.narrowPeak.gz
125	wgEncodeSydhTfbsK562Irf1Ifng6hStdPk.narrowPeak.gz
126	wgEncodeSydhTfbsK562Mafkab50322IggrabPk.narrowPeak.gz
127	wgEncodeSydhTfbsMcf10aesStat3Etoh01bStdPk.narrowPeak.gz
128	wgEncodeSydhTfbsMcf10aesStat3Etoh01cStdPk.narrowPeak.gz
129	wgEncodeSydhTfbsNb4MaxStdPk.narrowPeak.gz
130	wgEncodeSydhTfbsMcf10aesStat3TamStdPk.narrowPeak.gz
131	wgEncodeSydhTfbsNb4CmycStdPk.narrowPeak.gz
132	wgEncodeSydhTfbsMcf10aesStat3Etoh01StdPk.narrowPeak.gz
133	wgEncodeSydhTfbsPbdeGata1UcdPk.narrowPeak.gz
134	wgEncodeSydhTfbsShsy5yGata2UcdPk.narrowPeak.gz

3. The mathematical definition of evaluation criteria

3.1. Fisher's Exact Test score

Suppose that, given foreground dataset $\boldsymbol{F}$ ($|\boldsymbol{F}|=m$), background dataset $\boldsymbol{B}$ ($|\boldsymbol{B}|=n$), PWM $\mathbf{P}$ and a binding

energy threshold t. We count the number of sequences which contain **P** (its binding energy is greater than t), denoted as c_1, and the number of sequences which contain **P**, denoted as c_0. Then the Fisher Exact Test score is exactly the probability of obtaining at least c_1 sequences that contain **P** while selecting them with equal probabilities from ***F*** and ***B*** [8], formally we have

$$p = \sum_{k=c_1}^{\min(m, c_1+c_0)} \frac{\binom{m}{k}\binom{n}{c_1+c_0-k}}{\binom{m+n}{c_1+c_0}} \tag{13}$$

3.2. Minimal Hyper-Geometric score

Let $c_1(t)$ be the number of foreground sequences containing **P** under the threshold binding score t, and $c_0(t)$ be the number of background sequences containing **P**. Then the Minimal Hyper-Geometric score is defined as the minimum over all the possible t of the Fisher Exact Test scores[8]. Formally, we have

$$p = \min_t \sum_{k=c_1(t)}^{\min(m, c_1(t)+c_0(t))} \frac{\binom{m}{k}\binom{n}{c_1(t)+c_0(t)-k}}{\binom{m+n}{c_1(t)+c_0(t)}} \tag{14}$$

3.3. nCC and sASP

When launching a DMD finder on one dataset which the binding sites is known, we get a set of predicted binding sites. Following[9-11], we can obtain a contingency table and assess the prediction performance of tested method on this dataset both at the nucleotide level and at the site level with these two sets of binding messages,. Specifically, elements in the nucleotide level contingency table can be defined as follows:

- *nTP* is the number of nucleotide sites which are in both known sites set and predicted sites set;
- *nFN* is the number of nucleotide sites which are in known sites set but not in predicted sites set;
- *nFP* is the number of nucleotide sites which are not in known sites set but in predicted sites set;
- *nTN* is the number of nucleotide sites which are in neither known sites set nor predicted sites set.

We claim a predicted site success in identifying a known site if overlapping by at least one-quarter the length of the known site. Elements in the site level contingency table can be defined as follows:

- *sTP* be the number of known sites overlapped by predicted sites;
- *sFN* be the number of known sites not overlapped by predicted sites;
- *sFP* be the number of predicted sites not overlapped by known sites.

We define *Sensitivity* and *Positive Predictive Value* at either the nucleotide ($x = n$) or site ($x = s$) level as follows:

- *Sensitivity:* $xSn = xTP/(xTP + xFN)$;
- *Positive Predictive Value:* $xPPV = xTP/(xTP + xFP)$.

For nucleotide level we define two additional statistic:

- *Specificity:* $nSP = nTN/(nTN + nFP)$;
- *The (nucleotide level) correlation coefficient*

$$nCC = \frac{nTP \cdot nTN - nFN \cdot nFP}{\sqrt{(nTP+nFN)(nTN+nFP)(nTP+nFP)(nTN+nFN)}} \tag{15}$$

For site level we define the (site level) average site performance as

$$sASP = \frac{sSn + sPPV}{2} \qquad (16)$$

4. The construction of synthetic datasets

We first generated two sets of sequences as the foreground and background datasets, each set consists of 2000 500bp-long sequences that are sampled from a uniform distribution on DNA alphabets. For better mimicking real ChIP-seq data, three characteristics of TF-DNA interaction should be taken into account: (i) The motif of interest does not necessarily have an occurrence in all foreground sequences. (ii) The decoy motifs are pervasive in both foreground and background sequences. (iii) TFBS can be located either on the up or down strand. We therefore generated a signal motif PWM and a decoy motif PWM according to their settings of width and information content (IC) by choosing a random PWM and recursively polarizing it to archive a desired IC, respectively. Then, signal motifs and decoy motifs were implanted into two sets of selected sequences respectively. For signal motifs, the sequences were selected from foreground set with an implantation probability of 0.9, and for decoy motifs, the sequences were selected from both foreground and background set with a probability of 0.8. For each selected sequence one short sequence was sampled from corresponding target PWM (or its reverse complementary PWM) according to the sampling probability, and inserted into it on either up or down strand at a random non-overlapping position.

5. Additional performance comparison on synthetic datasets

5.1. Performance comparison on AUC, Fisher's exact test score and the Minimal Hyper-Geometric score for refinement test

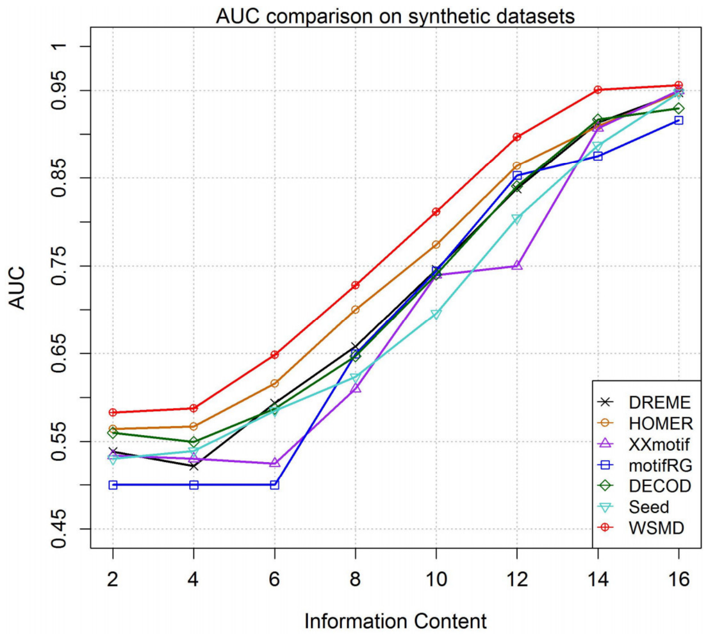

Fig. S3. The AUC comparison on refinement datasets for seeds and other six DMD tools.

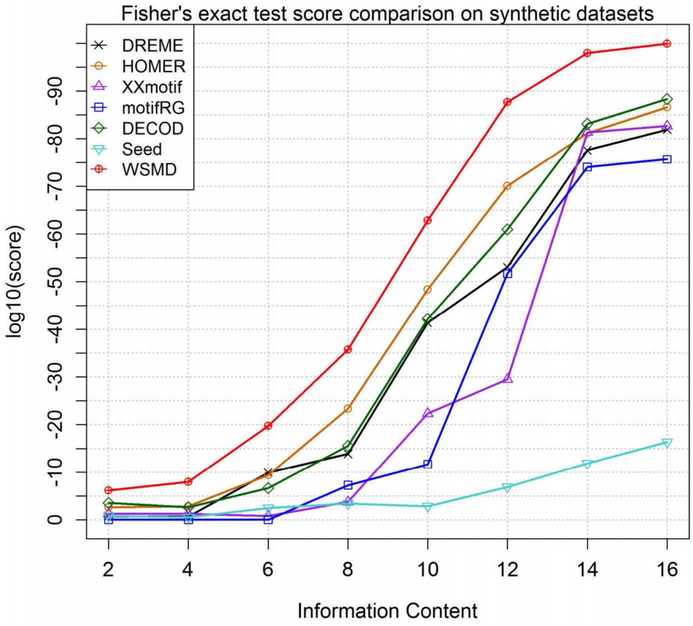

Fig. S4. The Fisher's exact test score comparison on refinement datasets for seeds and other six DMD tools.

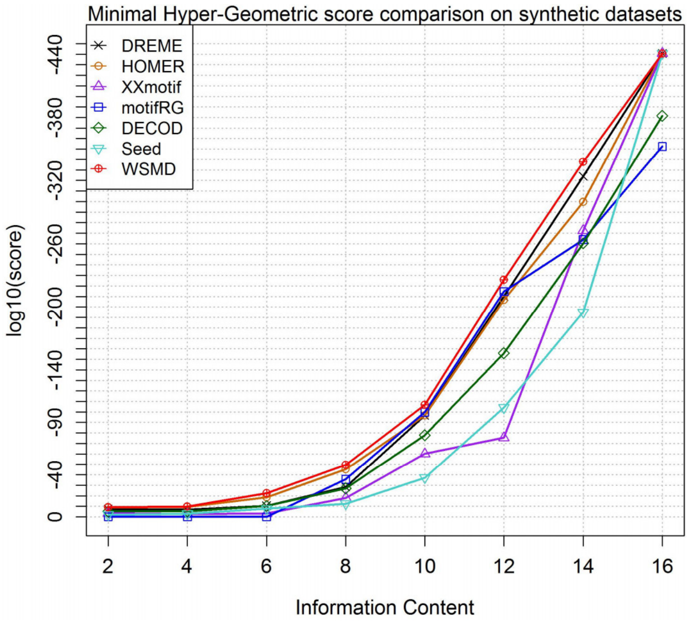

Fig. S5. The Minimal Hyper-Geometric score comparison on refinement datasets for seeds and other six DMD tools.

5.2. Performance comparison on AUC, Fisher's exact test score and the Minimal Hyper-Geometric score for extension test

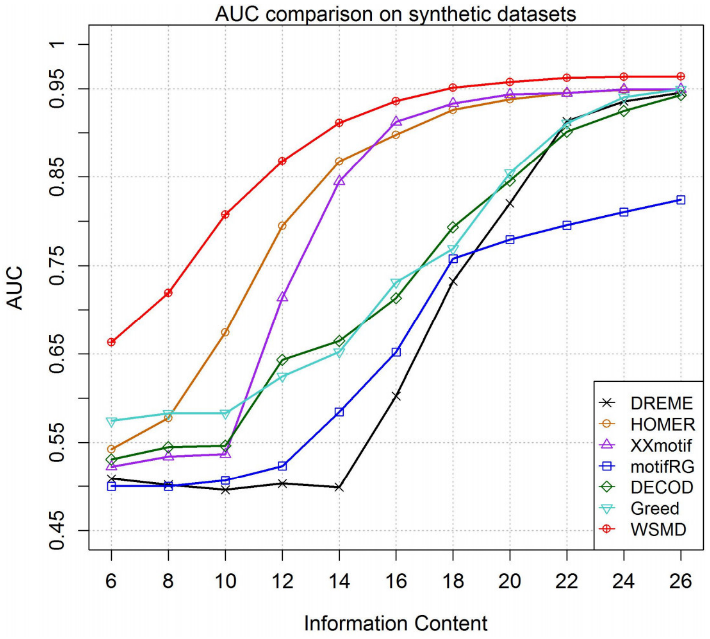

Fig. S6. The AUC comparison on extension datasets for Greed and other six DMD tools.

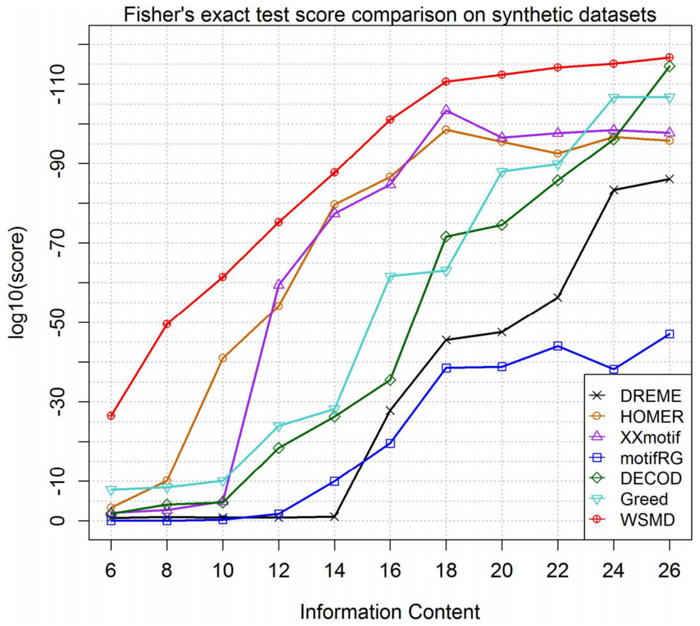

Fig. S7. The Fisher's exact test score comparison on extension datasets for Greed and other six DMD tools.

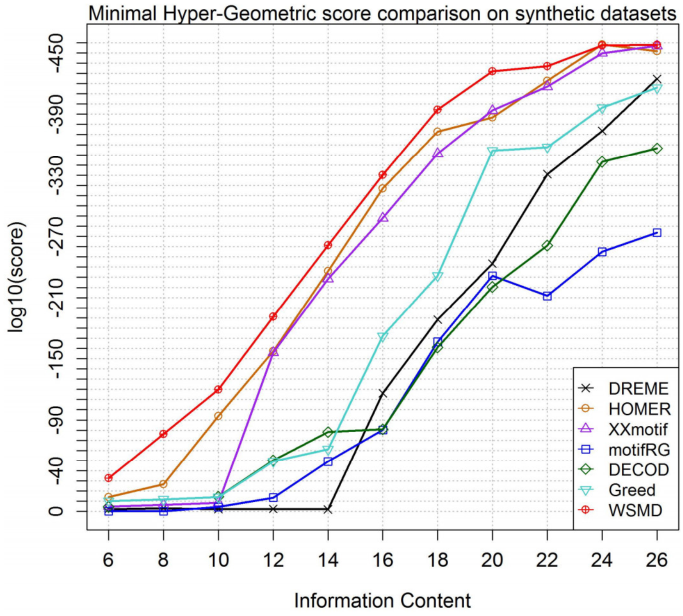

Fig. S8. The Minimal Hyper-Geometric score comparison on extension datasets for Greed and other five DMD tools.

6. Running time comparison

Table S2. Average running time on 10 datasets (seconds)

Sequence number	DREME	HOMER	XXmotif	motifRG	DECOD	WSMD
1000	15.35	8.635	264.78	88.19	131.12	12.48
2000	30.63	15.64	333.84	103.18	171.93	27.21
3000	50.83	22.96	592.68	110.75	182.39	33.92
4000	75.46	29.81	766.90	120.82	216.54	40.48
5000	93.42	35.78	1041.16	154.15	232.77	45.62
6000	103.36	42.05	1203.07	159.06	238.21	70.20
7000	125.90	49.49	1456.17	162.58	292.43	82.03
8000	136.73	56.45	1623.40	166.25	300.51	101.48
9000	155.37	62.83	1967.14	173.36	318.06	101.86
10000	170.71	68.86	2098.93	178.80	318.99	103.35

References

1 Yao, Z. *et al.* Discriminative motif analysis of high-throughput dataset. *Bioinformatics*, btt615 (2013).

2 Patel, R. Y. & Stormo, G. D. Discriminative motif optimization based on perceptron training. *Bioinformatics* **30**, 941-948 (2014).

3 Agostini, F., Cirillo, D., Ponti, R. D. & Tartaglia, G. G. SeAMotE: a method for high-throughput motif discovery in nucleic acid sequences. *BMC genomics* **15**, 925 (2014).

4 Fauteux, F., Blanchette, M. & Strömvik, M. V. Seeder: discriminative seeding DNA motif discovery. *Bioinformatics* **24**, 2303-2307 (2008).

5 Forsyth, D. Object Detection with Discriminatively Trained Part-Based Models. *Computer* **47**, 6-7 (2014).

6 Ren, W. Q., Huang, K. Q., Tao, D. C. & Tan, T. N. Weakly Supervised Large Scale Object Localization with Multiple Instance Learning and Bag Splitting. *Ieee Transactions on Pattern Analysis And Machine Intelligence* **38**, 405-416 (2016).

7 Cinbis, R. G., Verbeek, J. & Schmid, C. Weakly Supervised Object Localization with Multi-fold Multiple Instance Learning. *IEEE Transactions on Pattern Analysis & Machine Intelligence*, 1-1 (2015).

8 Tanaka, E., Bailey, T. L. & Keich, U. Improving MEME via a two-tiered significance analysis. *Bioinformatics* **30**, 1965-1973 (2014).

9 Tompa, M. *et al.* Assessing computational tools for the discovery of transcription factor binding sites. *Nature Biotechnology* **23**, 137-144 (2005).

10 Valen, E., Sandelin, A., Winther, O. & Krogh, A. Discovery of Regulatory Elements is Improved by a Discriminatory Approach. *Plos Computational Biology* **5**, e1000562-e1000562 (2009).

11 Hu, J., Yang, Y. D. & Kihara, D. EMD: an ensemble algorithm for discovering regulatory motifs in DNA sequences. *Bmc Bioinformatics* **7**, 1-13 (2006).
